# Do the body mass index and the diagnosis of gestational diabetes mellitus influence the level of physical activity during pregnancy and postpartum?

**DOI:** 10.1371/journal.pone.0220947

**Published:** 2019-08-09

**Authors:** Cibele de Oliveira Santini, Thiago dos Santos Imakawa, Geraldo Duarte, Silvana Maria Quintana, Elaine Christine Dantas Moisés

**Affiliations:** 1 Department of Gynecology and Obstetrics, Faculty of Medicine of Ribeirão Preto, University of São Paulo, Ribeirão Preto, São Paulo, Brazil; 2 Faculty of Medicine of Ribeirão Preto, University of São Paulo, Ribeirão Preto, São Paulo, Brazil; Monash University, AUSTRALIA

## Abstract

Obesity and lack of physical exercise are associated with the increase of diabetes mellitus in women of reproductive age and during the gestational period. The objective of the present study was to evaluate physical activity levels during the pregnancy and postpartum periods and the influence of body mass index (BMI) in women with gestational diabetes mellitus (GDM) or low risk pregnancy. The Pregnancy Physical Activity Questionnaire (PPAQ), translated and validated for Portuguese, was used for the evaluation of physical activity (PA) level. The sample was stratified according to preconception BMI and the presence or absence of diagnosis of GDM, resulting in four groups with 66 participants each: low risk pregnancy (LRP) with normal weight (BMI ≥ 18.5 and ≤ 24 kg/m^2^), LRP and overweight/obese (BMI ≥ 25 kg/m^2^), GDM with normal weight and GDM with overweight/obese. The level of PA of each participant was measured as Metabolic Equivalent of Task (MET) during the preconceptional period (T0), in the third trimester of gestation (T1), and three months after delivery (T2). The comparison of the MET values showed that the values found in the evaluation three months after delivery (T2) were higher than 1.00 (1.10 MET for the LRP-normal weight, 1.06 MET for LRP-overweight/obese, 1.02 MET for the GDM- normal weight, 1.07 MET for the GDM-overweight/obese). On the pre-gestational (T0) and third trimester (T1) analyzes, the values were less than 1.00 MET. The analysis between groups in relation to BMI and diagnosis of GDM showed no difference.

## Introduction

Lack of physical exercise associated with unhealthy diet contributes to obesity because they cause a change in body composition and alter the proportions of insulin receptors in muscles and adipose tissue. The prioritization of insulin action on adipocyte receptors leads to lower glucose uptake than to muscle receptors and consequently hyperglycemia, increased production of pancreatic insulin, and decreased body sensitivity to insulin [1]. Thus, the lack of regular exercise and obesity have been associated in the last decades to the increase in the prevalence of diabetes mellitus in the world population and, consequently, in women of reproductive age and during pregnancy. [2–4].

In 2015, the global prevalence of hyperglycemia first detected at any time during pregnancy was 16.9%, corresponding to 21.4 million exposed live-born infants [5]. In view of the severity of maternal-fetal and perinatal complications resulting from hyperglycemia [6,7], this metabolic disorder should be prevented or early controlled, with regular physical activity being one of the main strategies [8].

As a result of the several proven benefits, physical activity (PA) during pregnancy is usually recommended by different institutions, except in situations in which there are contraindication due to obstetric or clinical causes. The recommendation is a minimum of 30 minutes of moderate PA at least five times per week or totaling 150 minutes per week, avoiding intervals of more than 2 consecutive days without exercise [9–13]. However, despite the consolidated recommendations of exercise during pregnancy, a reduction in exercise levels are frequently observed during this period [8,12,14–20]. For example, in the United States a study showed that sixty percent of women reported not engaging in leisure time physical activity, and even for those who reported exercising regularly, physical activity progressively decreased by trimester [21].

Considering the evident need to elaborate and integrate exercise programs in prenatal care that combine effectiveness and greater adherence of the pregnant population, it is essential to estimate the level of PA in the population at low and intermediate obstetric risk, as well as to identify the variables that contribute to this scenario. The objective of the present study was to evaluate the influence of body mass index (BMI) in women with GDM on PA levels during gestational and postpartum periods.

## Patients and methods

The study was approved by the Ethics Committee of the University Hospital of the Ribeirão Preto Medical School, University of São Paulo (HCFMRP-USP) (Approval No. 1.358.154) and did not interfere with the obstetric management adopted for the selected patients. We selected women ≥ 18 years, literate, with singleton pregnancies and gestational age > 32 weeks who had no contraindication to exercise and who were under low-risk prenatal follow-up, defined as women without any pathology except overweight or obesity, or were diagnosed with GDM at the Women’s Health Referral Center of Ribeirão Preto—MATER or at HCFMRP-USP between January 2016 and February 2017.

**Fig 1:**
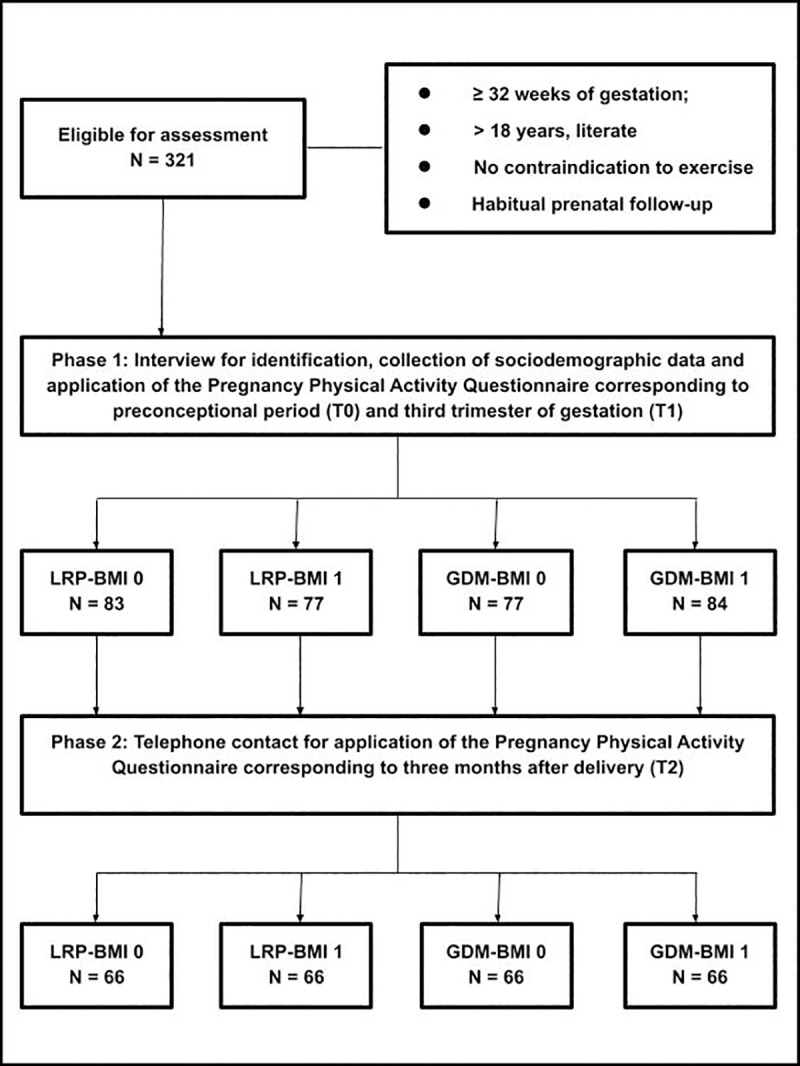
Flowchart of the study casuistry. LRP = low-risk pregnancy; GDM = gestational diabetes mellitus; BMI 0 = normal weight body mass index; BMI 1 = overweight/obesity.

The pregnant women who accepted to participate in the study were divided into four groups according to gestational risk and BMI (normal weight BMI: ≥ 18.5 and ≤ 24.9 kg/m^2^; overweight/obese BMI: ≥ 25 kg/m^2^): low-risk pregnancy and normal weight BMI (LRP-BMI 0); low-risk pregnancy and overweight/obese BMI (LRP-BMI 1); GDM and normal weight BMI (GDM-BMI 0); GDM and overweight/obese BMI (GDM-BMI 1) (Fig 1). We selected the groups by convenience sampling methods. Each group should have at least 66 patients, considering a level of significance of 5% and power of the test of 80%.

After inclusion and signing the free informed consent form, face-to-face interviews were held with the pregnant women to collect data such as maternal age, skin color, years of schooling, alcohol consumption, smoking, and self-reported preconception weight and height for characterization of the sample. The socioeconomic status of the patients was evaluated using the Brazilian Economic Classification Criteria [22] (Fig 1).

Physical activity was evaluated using the Pregnancy Physical Activity Questionnaire (PPAQ) [23], translated and validated for the Portuguese language by Silva et al [24]. The responses to the items of the questionnaire indicate the average time spent in each PA domain (in minutes or hours): leisure, household, caregiving, sports and exercise, transportation, and occupation. The women answered the PPAQ indicating the responses regarding the time spent that best fitted their activities during the preconception period (T0—referred about one month before pregnancy) and on the occasion of the interview (last trimester = T1). No instructions about additional PA were given during the interview to avoid interference with the prenatal follow-up of the patients. Three months after delivery (T2), the patients were contacted by telephone (data obtained from the hospital records) and responded again to the questionnaire for the period in question (Fig 1).

The level of PA was determined as metabolic equivalent (MET), which is defined as the energy expenditure at rest. MET is considered equivalent to oxygen consumption or VO_2_ (1 MET = 3.5 ml O_2_/kg/min) and to energy expenditure (1 MET = 1 kcal/kg/h) [25]. This resting value is used to quantify other activities, with each activity being expressed as a multiple of the resting metabolic rate [25]. Each activity is classified according to its intensity as sedentary (< 1.5 METs), light (1.5–2.9 METs), moderate (3.0–6.0 METs), or vigorous (> 6.0 METs) [23, 26, 27].

Differences in the qualitative variables between groups were determined by the chi-squared test and in the quantitative variables by analysis of variance. Tukey’s post-test was applied when necessary. A mixed effects linear regression model was fitted to compare the groups at each time point and time points in each group. The analysis was stratified by BMI and residual analysis using normality and dispersion graphs was performed for adjustment of the model. MET was the dependent variable and time and group were the independent variables. The model was implemented in the SAS 9.3 program using the PROC MIXED procedure.

## Results

The flowchart (Fig 1) shows the number of pregnant women in each phase of the study as well as the inclusion criteria. The initially calculated sample size was 264 patients, but the number of patients in each group was increased on average by 20% because of possible follow-up losses three months after delivery when the patients were contacted by telephone call. Thus, the initial sample consisted of 321 patients that answered the PPAQ regarding preconceptional and pregnancy periods, including 77 patients in LRP-BMI 1, 77 in GDM-BMI 0, 83 in LRP-BMI 0, and 84 in GDM-BMI 1. As a result of the loss to follow-up due to the impossibility of telephone contact after 10 attempts at different periods of the day during one week, the final sample for data collection by telephone contact 3 months after delivery consisted of 264 patients, with 66 patients per group.

The mean age (± standard deviation) of pregnant women with GDM was 26.55 (± 6.2) years in the BMI 0 group and 30.25 (± 6.03) years in the BMI 1 group, while the mean age of LRP women was 24.8 (± 5.16) and 27.52 (± 5.75) years in the BMI 0 and BMI 1 groups, respectively (Table 1). The mean maternal age was significantly higher in the GDM-BMI 1 group when compared to the GDM-BMI 0 and LRP-BMI 0 groups.

**Table 1:** Maternal clinical and sociodemographic characteristics (N = 321).

Variable		Group				
		LRP		GDM		
		BMI 0 (N = 83)	BMI 1 (N = 77)	BMI 0 (N = 77)	BMI 1 (N = 84)	P
Age and gestational history	Maternal age	24.80 (±5.16)	27.52 (±5.75)	26.55 (±6.20)	30.25 (±6.03)	<0.0001⁑
	No. of pregnancies	2.02 (±1.22)	2.34 (±1.26)	2.34 (±1.53)	2.49 (±1.59)	0.184⁑
	Parity	0.86 (±1.12)	1.16 (±1.08)	1.12 (±1.33)	1.18 (±1.40)	0.1823⁑
	Abortions	0.19 (±0.48)	0.18 (±0.51)	0.22 (±0.62)	0.31 (±0.54)	0.124⁑
Habits (N, %)	Smoking	6 (7.79%)	4 (5.33%)	5 (7.35%)	5 (6.10%)	0.926^☼^
	Alcohol consumption	1 (1.30%)	0 (0%)	1 (1.47%)	0 (0%)	0.5327^☼^
Skin color (N, %)	Non-white	33 (39.76%)	36 (46.75%)	35 (45.45%)	31 (36.90%)	0.5406^☼^
	White	50(60.24%)	41 (53.25%)	42 (54.55%)	53 (63.10%)	
Years of schooling (N, %)	< 8	7 (8.43%)	10 (12.99%)	13 (16.88%)	17 (20.24%)	
	= 8	12 (14.46%)	9 (11.69%)	11 (14.29%)	5 (5.95%)	
	> 8 and < 11	21 (25.30%)	17 (22.08%)	22 (28.57%)	13 (15.48%)	0.073^☼^
	= 11	42 (50.60%)	39 (50.65%)	27 (35.06%)	42 (50%)	
	> 12	1 (1.20%)	2 (2.60%)	4 (5.19%)	7 (8.33%)	
Household income, US$ (N, %)	6,297.07	0	0	1 (1.30%)	0	
	2,758.58	0	0	0	3 (3.61%)	0.1791^☼^
	1,374.96	10 (12.20%)	13 (16.88%)	11 (14.29%)	15 (18.07%)	
	748.14	25 (30.49%)	24 (31.17%)	16 (20.78%)	25 (30.12%)	
	449.15	36 (43.90%)	30 (38.96%)	31 (40.26%)	26 (31.33%)	
	198.70	11 (13.41%)	10 (12.99%)	18 (23.38%)	14 (16.87%)	
Route of delivery (N, %)	Cesarean	18 (23.38%)	28 (37.33%)	20 (29.41%)	34 (41.46%)	0.0751^☼^
	Vaginal	59 (76.62%)	47 (62.67%)	48 (70.59%)	48 (58.54%)	

LRP-BMI 0 = low-risk pregnancy and normal weight body mass index (≥ 18.5 and ≤ 24.9 kg/m^2^); LRP-BMI 1 = low-risk pregnancy and overweight/obese body mass index (≥ 25 kg/m^2^); GDM-BMI 0 = gestational diabetes mellitus and normal weight body mass index (≥ 18.5 and ≤ 24.9 kg/m^2^); ^2^; GDM-BMI 1 = gestational diabetes mellitus and overweight/obese body mass index (≥ 25 kg/m^2^); SD = standard deviation.

^☼^Chi-squared test

⁑Variance analysis

The number of pregnancies ranged from 2.02 to 2.49 (standard deviation of 1.22 to 1.59), with no difference between groups. The groups were homogenous in terms of the mean number of abortions, parity, alcohol consumption, and smoking (Table 1).

In all groups, most women belonged to socioeconomic class C2, which corresponds to a household income of US$ 449.15 (GDM-BMI 0 = 40.26%; GDM-BMI 1 = 31.33%; LRP-BMI 0 = 43.9%; LRP-BMI 1 = 38.96%). The second most prevalent socioeconomic class was C1, which corresponds to a household income of US$ 748.14 (20.78%, 30.12%, 30.49% and 31.17%, respectively) (Table 1).

The time factor was the most important parameter in the assessment of PA. Higher levels of PA were observed in the postpartum period when compared to the preconceptional and pregnancy periods, regardless of BMI stratification and identification of GDM (Table 2). Mean MET values > 1 were observed in all groups 3 months after delivery (T2) (1.10 MET for GDM-BMI 0 and 1.06 MET for GDM-BMI 1; 1.02 MET for LRP-BMI 0 and 1.07 MET for LRP-BMI 1). In contrast, MET values < 1 were calculated from the information reported by the patients in reference to the preconceptional period (T0) and in the third trimester (T1) (Table 2).

**Table 2:** Distribution of metabolic equivalent (MET) at each time point in the different groups according to body mass index category (N = 321).

BMI	Group	Time	N	Mean ±SD	Median (Q1 –Q3)	Min–Max
Normal weight (18,5–24,9 Kg/m^2^)	LRP	0	83	0.82 ± 0.46	0.68 (0.49–1.15)	0.10–2.27
		1	83	0.65 ± 0.35	0.57 (0.38–0.82)	0.11–1.55
		2	66	1.02 ± 0.26	1.01 (0.91–1.15)	0.17–1.86
	GDM	0	77	0.81 ± 0.44	0.75 (0.49–1.06)	0.06–2.05
		1	77	0.69 ± 0.42	0.56 (0.39–0.94)	0.08–2.01
		2	66	1.10 ± 0.35	1.06 (0.94–1.21)	0.40–2.30
Overweight/ obesity (≥ 25 Kg/m^2^)	LRP	0	77	0.99 ± 0.42	1.02 (0.70–1.25)	0.14–1.95
		1	77	0.76 ± 0.36	0.71 (0.46–1.03)	0.15–1.66
		2	66	1.07 ± 0.29	1.11 (0.87–1.27)	0.49–1.93
	GDM	0	84	0.80 ± 0.42	0.78 (0.46–1.02)	0.15–2.09
		1	84	0.71 ± 0.37	0.61 (0.44–0.90)	0.17–2.04
		2	66	1.06 ± 0.36	1.05 (0.81–1.24)	0.27–1.88

BMI = body mass index; LRP = low-risk pregnancy; GDM = gestational diabetes mellitus; time 0 = preconceptional; time 1 = third trimester of gestation; time 2 = 3 months after delivery; N = number of pregnant women; SD = standard deviation; Q1 = first quartile; Q3 = third quartile; Min = minimum; Max = maximum.

Distribution of quantitative variables in relation to the groups.

Comparison of time points in GDM-BMI 0 and GDM-BMI 1 showed a difference in mean METs at T2 when compared to T1 and the same difference was observed in mean METs at T2 when compared with to T0. Comparing the mean METs presented by the LRP-BMI group 0, a difference was observed between the three moments evaluated. In LRP-BMI 1, it was found difference between T2 and T1 and between T1 and T0 (Table 3). Finally, comparison of the GDM and LRP groups with overweight/obese BMI showed lower mean METs in the former at T0 (Table 3).

**Table 3:** Estimated difference between means for comparisons between time points and between groups in each body mass index category (N = 321).

BMI	Comparison	Estimated difference between means	95% CI		P
Normal Weight (18.5 to 24.9 kg/m^2^)	(T0–T1) GDM	0.1216	0.03371	0.2096	0.0069
	(T0–T2) GDM	-0.2962	-0.3889	-0.2036	<0.0001
	(T1–T2) GDM	-0.4179	-0.5106	-0.3252	<0.0001
	(T0–T1) LRP	0.1778	0.09306	0.2624	<0.0001
	(T0–T2) LRP	-0.2136	-0.3056	-0.1216	<0.0001
	(T1–T2) LRP	-0.3914	-0.4833	-0.2994	<0.0001
	T0 (GDM—LRP)	-0.01339	-0.1353	0.1085	0.829
	T1 (GDM—LRP)	0.04272	-0.07917	0.1646	0.4909
	T2 (GDM—LRP)	0.06924	-0.06092	0.1994	0.296
Overweight/ obesity (≥ 25 kg/m^2^)	(T0–T1) GDM	0.08677	0.002436	0.1711	0.0438
	(T0–T2) GDM	-0.2729	-0.3645	-0.1814	<0.0001
	(T1–T2) GDM	-0.3597	-0.4513	-0.2681	<0.0001
	(T0–T1) LRP	0.2305	0.1422	0.3188	<0.0001
	(T0–T2) LRP	-0.06306	-0.1561	0.02996	0.1831
	(T–T2) LRP	-0.2936	-0.3863	-0.2008	<0.0001
	T0 (GDM—LRP)	-0.1960	-0.3127	-0.07935	0.0011
	T1 (GDM—LRP)	-0.05229	-0.1690	0.06441	0.3786
	T2 (GDM—LRP)	0.01386	-0.1116	0.1393	0.828

GDM = gestational diabetes mellitus; LRP = low-risk pregnancy; BMI = body mass index; 95% CI = 95% confidence interval; T0 = preconceptional; T1 = third trimester of gestation; T2 = 3 months after delivery.

Mixed effects regression model.

## Discussion

Comparison of time points showed a difference in METs postpartum compared to the third trimester of pregnancy and preconceptional periods in women with gestational diabetes mellitus and normal weight BMI and in women with gestational diabetes mellitus and overweight/obese BMI. On the other hand, in women with low-risk pregnancy and normal weight BMI, a difference in mean METs was observed between all time points. In women with low-risk pregnancy and overweight/obese BMI, a difference was found between pospartum and the third trimester of gestation periods and between this period and preconceptional period. This increase in mean METs over the period of 3 months after delivery might be due to the care demands of the newborn, which remain more intense up to 12 months after birth [28, 29]. It is emphasized that, regardless of the differences observed in intra and intergroup analysis, from a clinical point of view, no change in PA level was observed, with all participants continuing to be classified as sedentary (MET < 1.5) [27].

There is considerable heterogeneity in the classification and methods used to evaluate PA level. This controversy explains the differences found in the prevalence of sedentarism and impairs the comparison between studies involving different populations [30].

In contrast to the present study, other authors reported a reduction in PA level after birth due to the existence of personal and environmental barriers to exercise and sports. Personal factors include the influence of household income, care with other children, lack of a partner during exercise, and lack of exercise support from family [19, 31–33]. Another important personal factor are the high care needs of the newborn, highlighted in other studies as impairing the participation in sports and regular exercises and contributing to lower PA levels. However, it should be noted that the populations studied had higher initial PA levels than the sample of the present study [34]. Environmental factors that contribute to exercising less include those that cannot be controlled by the mother, such as access to public transportation, safe leisure-time facilities, and lack of a health information system [34].

The sedentary PA level is usually more common among individuals from lower socioeconomic classes [35]. The sample of the present study was predominantly composed of patients of socioeconomic class with a family income of approximately US $ 449.15, corresponding to 41.88% in the women with low-risk pregnancy group and to 35.63% in the women with gestational diabetes mellitus group. This finding might explain the predominance of sedentary level observed. A study conducted in Pelotas, Brazil, which involved more than 5,000 young adults who responded to the short version of the International Physical Activity Questionnaire, showed a lower than expected increase in PA rates from 41.1% in 2002 to 52.0% in 2007 in the low-income population. The responsible factors for decrease of physical activity would be industrial mechanization and a consequent decrease in human activity (considering that low-income workers tend to participate in activities related to manual labor), increased use of public transportation, as well as an increase in purchasing power which permits the acquisition of motorcycles and cars, thus reducing transport-related activity [36].

Alarming rates of sedentary PA level are observed in different countries. In the United States, children and adults spend approximately 55% of their waking hours (7.7 hours/day) in activities that result in very low levels of energy expenditure. This estimate reaches almost 60% (more than 8 hours/day) in adolescents and adults between 60 and 85 years of age. Regarding gender, women are more sedentary than men [37].

The education level is directly related to a reduction in PA level. Pregnant women with higher education spend fewer hours in sedentary activities than those who only have a high school diploma [38]. Data from the Brazilian Institute of Geography and Statistics have shown that the larger the number of years of schooling, the higher the percentage of individuals practicing some sport [38]. In addition to education level, the socioeconomic class seems to influence the participation in PA and sports, which is more widespread in classes of higher monthly household income per capita [39, 40]. Taken together, our results of low PA levels are compatible with the low education level of the patients.

Specifically for leisure-time PA, studies also suggest an association between the high prevalence of inactivity and sociodemographic indicators [41]. The lower the education level and household income, the lower the chance of engagement in leisure-time PA because of the lack of social and environmental resources and information [42, 43].

With respect to the impact on physical health, exercise is related to the prevention and treatment of diseases such as GDM, gestational hypertension and obesity [44–48], while sedentarism results in a significant increase in health expenditures [49,50]. Specifically for diabetes, an important aspect is the economic impact caused by the disease and its complications, with substantial economic loss to patients and their families, as well as to health systems and national economies [4]. This economic burden is expressed directly as medical costs and is indirectly associated with the loss of productivity, premature mortality and a negative impact on the nation’s gross domestic product [4]. In 2011, the Harvard School of Public Health reported global gross domestic product losses due to direct and indirect costs from diabetes totaling US$ 1.7 trillion [51].

The homogenous finding of sedentary PA level in the sample studied contrasts with the consolidated concepts of the relevant benefits of PA during pregnancy and postpartum for the mother-fetus and newborn [44, 52–55], but is compatible with the global epidemiological scenario. A recent review demonstrated that pregnant women spend more than 50% of their time (57.1 to 78%) in sedentary activities, i.e., those using less than 1.5 MET [30].

Despite these data, programs or booklets with detailed information and instructions that incorporate exercises in the routine of the pregnant population are sparse in Brazil. It is therefore necessary that health managers and healthcare workers from the private and public sector elaborate exercise programs for all pregnant women [46].

This study presented some limitations to be considered. This was an observational study with a casuistry composed of women belonging to socioeconomic stratification predominantly with shorter schooling time and low family income, with no representation of all socioeconomic classes. It should be emphasized that the instrument used is characterized by a subjective measure in the form of a questionnaire, which was not compared with a direct physical activity measures, which are considered to present better precision.

## Conclusion

In the present study, neither BMI nor a diagnosis of GDM interfered with the PA level of pregnant women. On the other hand, the time factor, specifically the postpartum period, determined higher PA levels when compared to preconception and pregnancy in both women with low-risk pregnancy and women with a diagnosis of GDM. Despite the statistical difference, there was no clinical relevance in the MET's difference because the patients still remained on the sedentary physical activity level according to PPAQ.

In view of the growing sedentary PA level in the population studied, the importance of exercise for the prevention and treatment of GDM and the severe complications of hyperglycemia, the development of public health policies providing safer instructions and exercise programs for pregnant women should be reinforced.

## Supporting information

S1 Appendix Original pregnancy physical activity questionnaire. Reproduced from Chasan-Taber L, Schmidt MD, Roberts DE, Hosmer D, Markenson G, Freedson PS. Development and Validation of a Pregnancy Physical Activity Questionnaire. Med Sci Sports Exerc. 2004; 36(10): 1750–60.
(PDF)

S2 Appendix Brazilian portuguese translation of the pregnancy physical activity questionnaire. (PDF)

S3 Appendix Socioeconomic questionnaire. Brazilian economic Classification Criteria from Associação Brasileira de Empresas e Pesquisas (Brazilian Association of Companies and Research).
(PDF)

S1 Dataset Study dataset. L Time 0: preconceptional period; Time 1: third trimester of gestation; Time 2: three months after delivery; GDM: gestational diabetes mellitus; LRP: low-risk pregnancy; BMI: body mass index; Normal weight (≥ 18,5 and ≤ 24,9 Kg/m^2^); Overweight/Obese (≥ 25 Kg/m^2^); MET: metabolic equivalent of task.
(XLSX)
