# Supplementary material for: Do the body mass index and the diagnosis of gestational diabetes mellitus influence the level of physical activity during pregnancy and postpartum?
Source: PLoS One. 2019 Aug 9;14(8):e0220947. doi: 10.1371/journal.pone.0220947 (PMC6688803; doi:10.1371/journal.pone.0220947)

Pregnancy Physical Activity Questionnaire

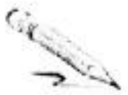

Instructions:
Please use an ordinary No. 2 pencil. *Fill in the circles completely. The Question will be read by a machine so if you need to change your answer, erase the incorrect mark* ***completely****. If you have comments, please write them on the back of the questionnaire.*

Example: During this trimester, when you are NOT at work, how much time do you usually spend:

E1. **Taking care of an older adult**

If you take care of your mom for 2 hours each day, then your answer should look like this... →

- ○ None
- ○ Less than 1/2 hour per day
- ○ 1/2 to almost 1 hour per day
- ○ 1 to almost 2 hours per day
- ● 2 to almost 3 hours per day
- ○ 3 or more hours per day

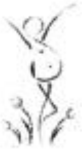

It is very important you tell us about yourself honestly. There are no right or wrong answers. We just want to know about the things you are doing during this trimester.

1. Today's Date: ☐☐ / ☐☐ / ☐☐☐☐ (Month / Day / Year)
2. What was the first day of your last period? ☐☐ / ☐☐ / ☐☐☐☐ (Month / Day / Year) ○ I don't know
3. When is your baby due? ☐☐ / ☐☐ / ☐☐☐☐ (Month / Day / Year) ○ I don't know

---

During this trimester, when you are NOT at work, how much time do you usually spend:

4. **Preparing meals (cook, set table, wash dishes)**
   - ○ None
   - ○ Less than 1/2 hour per day
   - ○ 1/2 to almost 1 hour per day
   - ○ 1 to almost 2 hours per day
   - ○ 2 to almost 3 hours per day
   - ○ 3 or more hours per day

5. **Dressing, bathing, feeding children while you are <u>sitting</u>**
   - ○ None
   - ○ Less than 1/2 hour per day
   - ○ 1/2 to almost 1 hour per day
   - ○ 1 to almost 2 hours per day
   - ○ 2 to almost 3 hours per day
   - ○ 3 or more hours per day

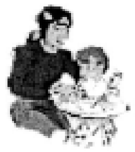

6. **Dressing, bathing, feeding children while you are <u>standing</u>**
   - ○ None
   - ○ Less than 1/2 hour per day
   - ○ 1/2 to almost 1 hour per day
   - ○ 1 to almost 2 hours per day
   - ○ 2 to almost 3 hours per day
   - ○ 3 or more hours per day

7. **Playing with children while you are <u>sitting or standing</u>**
   - ○ None
   - ○ Less than 1/2 hour per day
   - ○ 1/2 to almost 1 hour per day
   - ○ 1 to almost 2 hours per day
   - ○ 2 to almost 3 hours per day
   - ○ 3 or more hours per day

8. **Playing with children while you are <u>walking or running</u>**
   - ○ None
   - ○ Less than 1/2 hour per day
   - ○ 1/2 to almost 1 hour per day
   - ○ 1 to almost 2 hours per day
   - ○ 2 to almost 3 hours per day
   - ○ 3 or more hours per day

9. **Carrying children**

- ○ None
- ○ Less than 1/2 hour per day
- ○ 1/2 to almost 1 hour per day
- ○ 1 to almost 2 hours per day
- ○ 2 to almost 3 hours per day
- ○ 3 or more hours per day

10. **Taking care of an older adult**

- ○ None
- ○ Less than 1/2 hour per day
- ○ 1/2 to almost 1 hour per day
- ○ 1 to almost 2 hours per day
- ○ 2 to almost 3 hours per day
- ○ 3 or more hours per day

11. **Sitting and using a computer or writing, while <u>not</u> at work**

- ○ None
- ○ Less than 1/2 hour per day
- ○ 1/2 to almost 1 hour per day
- ○ 1 to almost 2 hours per day
- ○ 2 to almost 3 hours per day
- ○ 3 or more hours per day

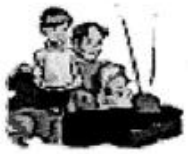

12. **Watching TV or a video**

- ○ None
- ○ Less than 1/2 hour per day
- ○ 1/2 to almost 2 hours per day
- ○ 2 to almost 4 hours per day
- ○ 4 to almost 6 hours per day
- ○ 6 or more hours per day

13. **Sitting and reading, talking, or on the phone, while <u>not</u> at work**

- ○ None
- ○ Less than 1/2 hour per day
- ○ 1/2 to almost 2 hours per day
- ○ 2 to almost 4 hours per day
- ○ 4 to almost 6 hours per day
- ○ 6 or more hours per day

14. **Playing with pets**

- ○ None
- ○ Less than 1/2 hour per day
- ○ 1/2 to almost 1 hour per day
- ○ 1 to almost 2 hours per day
- ○ 2 to almost 3 hours per day
- ○ 3 or more hours per day

15. **Light cleaning (make beds, laundry, iron, put things away)**

- ○ None
- ○ Less than 1/2 hour per day
- ○ 1/2 to almost 1 hour per day
- ○ 1 to almost 2 hours per day
- ○ 2 to almost 3 hours per day
- ○ 3 or more hours per day

16. **Shopping (for food, clothes, or other items)**

- ○ None
- ○ Less than 1/2 hour per day
- ○ 1/2 to almost 1 hour per day
- ○ 1 to almost 2 hours per day
- ○ 2 to almost 3 hours per day
- ○ 3 or more hours per day

During this trimester, when you are NOT at work, how much time do you usually spend:

17. **Heavier cleaning (vacuum, mop, sweep, wash windows)**

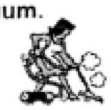

- ○ None
- ○ Less than 1/2 hour per week
- ○ 1/2 to almost 1 hour per week
- ○ 1 to almost 2 hours per week
- ○ 2 to almost 3 hours per week
- ○ 3 or more hours per week

18. **Mowing lawn while on a riding mower**

- ○ None
- ○ Less than 1/2 hour per week
- ○ 1/2 to almost 1 hour per week
- ○ 1 to almost 2 hours per week
- ○ 2 to almost 3 hours per week
- ○ 3 or more hours per week

19. **Mowing lawn using a walking mower, raking, gardening**

- ○ None
- ○ Less than 1/2 hour per week
- ○ 1/2 to almost 1 hour per week
- ○ 1 to almost 2 hours per week
- ○ 2 to almost 3 hours per week
- ○ 3 or more hours per week

Going Places...

During this trimester, how much time do you usually spend:

20. **Walking <u>slowly</u> to go places (such as to the bus, work, visiting)**
<u>Not</u> for fun or exercise

- ○ None
- ○ Less than 1/2 hour per day
- ○ 1/2 to almost 1 hour per day
- ○ 1 to almost 2 hours per day
- ○ 2 to almost 3 hours per day
- ○ 3 or more hours per day

21. **Walking quickly to go places (such as to the bus, work, or school)**
<u>Not</u> for fun or exercise

- ○ None
- ○ Less than 1/2 hour per day
- ○ 1/2 to almost 1 hour per day
- ○ 1 to almost 2 hours per day
- ○ 2 to almost 3 hours per day
- ○ 3 or more hours per day

22. **Driving or riding in a car or bus**

- ○ None
- ○ Less than 1/2 hour per day
- ○ 1/2 to almost 1 hour per day
- ○ 1 to almost 2 hours per day
- ○ 2 to almost 3 hours per day
- ○ 3 or more hours per day

For Fun or Exercise...

During this trimester, how much time do you usually spend:

23. **Walking slowly for fun or exercise**
   - O None
   - O Less than 1/2 hour per week
   - O 1/2 to almost 1 hour per week
   - O 1 to almost 2 hours per week
   - O 2 to almost 3 hours per week
   - O 3 or more hours per week

24. **Walking more quickly for fun or exercise**
   - O None
   - O Less than 1/2 hour per week
   - O 1/2 to almost 1 hour per week
   - O 1 to almost 2 hours per week
   - O 2 to almost 3 hours per week
   - O 3 or more hours per week

25. **Walking quickly up hills for fun or exercise**
   - O None
   - O Less than 1/2 hour per week
   - O 1/2 to almost 1 hour per week
   - O 1 to almost 2 hours per week
   - O 2 to almost 3 hours per week
   - O 3 or more hours per week

During this trimester, how much time do you usually spend:

26. **Jogging**
   - O None
   - O Less than 1/2 hour per week
   - O 1/2 to almost 1 hour per week
   - O 1 to almost 2 hours per week
   - O 2 to almost 3 hours per week
   - O 3 or more hours per week

27. **Prenatal exercise class**
   - O None
   - O Less than 1/2 hour per week
   - O 1/2 to almost 1 hour per week
   - O 1 to almost 2 hours per week
   - O 2 to almost 3 hours per week
   - O 3 or more hours per week

28. **Swimming**
   - O None
   - O Less than 1/2 hour per week
   - O 1/2 to almost 1 hour per week
   - O 1 to almost 2 hours per week
   - O 2 to almost 3 hours per week
   - O 3 or more hours per week

29. **Dancing**
   - O None
   - O Less than 1/2 hour per week
   - O 1/2 to almost 1 hour per week
   - O 1 to almost 2 hours per week
   - O 2 to almost 3 hours per week
   - O 3 or more hours per week

Doing other things for fun or exercise? Please tell us what they are.

30. ______________________
   Name of Activity
   - O None
   - O Less than 1/2 hour per week
   - O 1/2 to almost 1 hour per week
   - O 1 to almost 2 hours per week
   - O 2 to almost 3 hours per week
   - O 3 or more hours per week

31. ______________________
   Name of Activity
   - O None
   - O Less than 1/2 hour per week
   - O 1/2 to almost 1 hour per week
   - O 1 to almost 2 hours per week
   - O 2 to almost 3 hours per week
   - O 3 or more hours per week

Please fill out the next section if you work for wages, as a volunteer, or if you are a student. If you are a homemaker, out of work, or unable to work, you do not need to complete this last section.

At Work...

During this trimester, how much time do you usually spend:

32. **Sitting at working or in class**

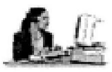

   - O None
   - O Less than 1/2 hours per day
   - O 1/2 to almost 2 hours per day
   - O 2 to almost 4 hours per day
   - O 4 to almost 6 hours per day
   - O 6 or more hours per day

33. **Standing or slowly walking at work while carrying things (heavier than a 1 gallon milk jug)**
   - O None
   - O Less than 1/2 hour per day
   - O 1/2 to almost 2 hours per day
   - O 2 to almost 4 hours per day
   - O 4 to almost 6 hours per day
   - O 6 or more hours per day

34. **Standing or slowly walking at work not carrying anything**
   - O None
   - O Less than 1/2 hours per day
   - O 1/2 to almost 2 hours per day
   - O 2 to almost 4 hours per day
   - O 4 to almost 6 hours per day
   - O 6 or more hours per day

35. **Walking quickly at work while carrying things (heavier than a 1 gallon milk jug)**
   - O None
   - O Less than 1/2 hour per day
   - O 1/2 to almost 2 hours per day
   - O 2 to almost 4 hours per day
   - O 4 to almost 6 hours per day
   - O 6 or more hours per day

36. **Walking quickly at work not carrying anything**
   - O None
   - O Less than 1/2 hour per day
   - O 1/2 to almost 2 hours per day
   - O 2 to almost 4 hours per day
   - O 4 to almost 6 hours per day
   - O 6 or more hours per day

Thank You

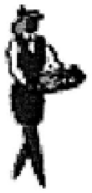

Supplement: S1 Appendix — Reproduced from Chasan-Taber L, Schmidt MD, Roberts DE, Hosmer D, Markenson G, Freedson PS. Development and Validation of a Pregnancy Physical Activity Questionnaire. Med Sci Sports Exerc. 2004; 36(10): 1750–60. (PDF) [file pone.0220947.s001.pdf]
