# Supplementary material for: Do the body mass index and the diagnosis of gestational diabetes mellitus influence the level of physical activity during pregnancy and postpartum?
Source: PLoS One. 2019 Aug 9;14(8):e0220947. doi: 10.1371/journal.pone.0220947 (PMC6688803; doi:10.1371/journal.pone.0220947)
Supplement: S2 Appendix — (PDF) [file pone.0220947.s002.pdf]

Questionário de Atividade Física para Gestantes – QAFG

É muito importante que você responda honestamente. Não há nenhuma resposta certa ou errada.

1. Qual foi o dia de sua última menstruação? ____/____/______ Eu não sei. Aproximadamente.

2. Quando o seu bebê vai nascer? ____/____/______ ☐ Eu não sei.

Quando você NÃO está no trabalho, quanto tempo, você geralmente passa:

3 Preparando as refeições (cozinhando, colocando a mesa, lavando os pratos).

			DATAS
**0**			Nenhum
**1**			Menos que 30 minutos por dia
**2**			De 30 minutos a 1 hora por dia
**3**			De 1 hora a 2 horas por dia
**4**			De 2 hora a 3 horas por dia
**5**			De 3 horas ou mais por dia

4 Cuidando de criança (Vestindo, banhando, Alimentando enquanto você fica sentada).

			DATAS
			Nenhum
			Menos que 30 minutos por dia
			De 30 minutos a 1 hora por dia
			De 1 hora a 2 horas por dia
			De 2 hora a 3 horas por dia
			De 3 horas ou mais por dia

5 Cuidando de criança (Vestindo, banhando, alimentando enquanto você está em pé).

			Nenhum
			Menos que 30 minutos por dia
			De 30 minutos a 1 hora por dia
			De 1 hora a 2 horas por dia
			De 2 hora a 3 horas por dia
			De 3 horas ou mais por dia

6 Brincando com criança enquanto você está Sentada ou em pé.

			Nenhum
			Menos que 30 minutos por dia
			De 30 minutos a 1 hora por dia
			De 1 hora a 2 horas por dia
			De 2 hora a 3 horas por dia
			De 3 horas ou mais por dia

7 Brincando com a criança enquanto você Está caminhando ou correndo.

			Nenhum
			Menos que 30 minutos por dia
			De 30 minutos a 1 hora por dia
			De 1 hora a 2 horas por dia
			De 2 hora a 3 horas por dia
			De 3 horas ou mais por dia

8 Carregando criança nos braços.

			Nenhum
			Menos que 30 minutos por dia
			De 30 minutos a 1 hora por dia
			De 1 hora a 2 horas por dia
			De 2 hora a 3 horas por dia
			De 3 horas ou mais por dia

9 Cuidando de idoso adulto, incapacitado.

			Nenhum
			Menos que 30 minutos por dia
			De 30 minutos a 1 hora por dia
			De 1 hora a 2 horas por dia
			De 2 hora a 3 horas por dia
			De 3 horas ou mais por dia

10 Sentada: usando o computador, lendo, escrevendo, ou falando ao telefone Não estando trabalhando.

			Nenhum
			Menos que 30 minutos por dia
			De 30 minutos a 1 hora por dia
			De 1 hora a 2 horas por dia
			De 2 hora a 3 horas por dia
			De 3 horas ou mais por dia

Quanto tempo, você geralmente passa:

11 Assistindo TV ou vídeo.

			Nenhum
			Menos que 30 minutos por dia
			De 30 minutos a 1 hora por dia
			De 1 hora a 2 horas por dia
			De 2 hora a 3 horas por dia
			De 3 horas ou mais por dia

12 Brincando com animais de estimação .

			Nenhum
			Menos que 30 minutos por dia
			De 30 minutos a 1 hora por dia
			De 1 hora a 2 horas por dia
			De 2 hora a 3 horas por dia
			De 3 horas ou mais por dia

13 Fazendo limpeza leve (arrumar as camas, Passar roupas, levar o lixo para fora)

			Nenhum
			Menos que 30 minutos por dia
			De 30 minutos a 1 hora por dia
			De 1 hora a 2 horas por dia
			De 2 hora a 3 horas por dia
			De 3 horas ou mais por dia

14 Fazendo compras (roupas, comidas ou outros objetos).

			Nenhum
			Menos que 30 minutos por dia
			De 30 minutos a 1 hora por dia
			De 1 hora a 2 horas por dia
			De 2 hora a 3 horas por dia
			De 3 horas ou mais por dia

15 Fazendo limpeza mais pesada (aspirar, varrer, esfregar o chão, lavar roupas, ou lavar janelas)

			Nenhum
			Menos que 30 minutos por dia
			De 30 minutos a 1 hora por dia
			De 1 hora a 2 horas por dia
			De 2 hora a 3 horas por dia
			De 3 horas ou mais por dia

16 Empurrando cortador de grama, apanhando folhas ou trabalhando no jardim.

			Nenhum
			Menos que 30 minutos por dia
			De 30 minutos a 1 hora por dia
			De 1 hora a 2 horas por dia
			De 2 hora a 3 horas por dia
			De 3 horas ou mais por dia

Indo a lugares...

Quanto tempo, você geralmente passa:

17 Caminhando lentamente para (pegar o ônibus, ir para trabalho ou fazer visitas)

			Nenhum
			Menos que 30 minutos por dia
			De 30 minutos a 1 hora por dia
			De 1 hora a 2 horas por dia
			De 2 hora a 3 horas por dia
			De 3 horas ou mais por dia

18 Caminhando rapidamente para (pegar o ônibus, ir para trabalho ou escola).

			Nenhum
			Menos que 30 minutos por dia
			De 30 minutos a 1 hora por dia
			De 1 hora a 2 horas por dia
			De 2 hora a 3 horas por dia
			De 3 horas ou mais por dia

19 Dirigindo, ou andando de carro ou de ônibus.

			Nenhum
			Menos que 30 minutos por dia
			De 30 minutos a 1 hora por dia
			De 1 hora a 2 horas por dia
			De 2 hora a 3 horas por dia
			De 3 horas ou mais por dia

Para diversão ou exercício....

Quanto tempo, você geralmente passa:

20 Caminhando lentamente por divertimento ou Exercício.

			Nenhum
			Menos que 30 minutos por dia
			De 30 minutos a 1 hora por dia
			De 1 hora a 2 horas por dia
			De 2 hora a 3 horas por dia
			De 3 horas ou mais por dia

21 Caminhando mais rápido, por divertimento ou exercício

			Nenhum
			Menos que 30 minutos por dia
			De 30 minutos a 1 hora por dia
			De 1 hora a 2 horas por dia
			De 2 hora a 3 horas por dia
			De 3 horas ou mais por dia

22 Caminhando mais rápido ladeira a cima, por Divertimento ou exercício.

			Nenhum
			Menos que 30 minutos por dia
			De 30 minutos a 1 hora por dia
			De 1 hora a 2 horas por dia
			De 2 hora a 3 horas por dia
			De 3 horas ou mais por dia

23 Fazendo “Cooper” (trote ou corrida moderada)

			Nenhum
			Menos que 30 minutos por dia
			De 30 minutos a 1 hora por dia
			De 1 hora a 2 horas por dia
			De 2 hora a 3 horas por dia
			De 3 horas ou mais por dia

24 Na aula de exercício pré natal.

			Nenhum
			Menos que 30 minutos por dia
			De 30 minutos a 1 hora por dia
			De 1 hora a 2 horas por dia
			De 2 hora a 3 horas por dia
			De 3 horas ou mais por dia

25 Nadando

			Nenhum
			Menos que 30 minutos por dia
			De 30 minutos a 1 hora por dia
			De 1 hora a 2 horas por dia
			De 2 hora a 3 horas por dia
			De 3 horas ou mais por dia

26 Dançando

			Nenhum
			Menos que 30 minutos por dia
			De 30 minutos a 1 hora por dia
			De 1 hora a 2 horas por dia
			De 2 hora a 3 horas por dia
			De 3 horas ou mais por dia

Fazendo outras coisas por divertimento ou exercício? Por favor, relacionar as atividades:

27 ______________________________
nome da atividade

			Nenhum
			Menos que 30 minutos por dia
			De 30 minutos a 1 hora por dia
			De 1 hora a 2 horas por dia
			De 2 hora a 3 horas por dia
			De 3 horas ou mais por dia

28 ______________________________
nome da atividade

			Nenhum
			Menos que 30 minutos por dia
			De 30 minutos a 1 hora por dia
			De 1 hora a 2 horas por dia
			De 2 hora a 3 horas por dia
			De 3 horas ou mais por dia

Só preencha a próxima etapa se a gestante trabalha por salário, como voluntária ou se for estudante. Se for dona de casa, desempregada, ou incapacitada de trabalhar, você não precisa preencher essa etapa.

No Trabalho....

Quanto tempo, você geralmente passa:

29 Sentada, trabalhando em sala de aula ou escritório.

			Nenhum
			Menos que 30 minutos por dia
			De 30 minutos a 1 hora por dia
			De 1 hora a 2 horas por dia
			De 2 hora a 3 horas por dia
			De 3 horas ou mais por dia

30 Em pé ou caminhando lentamente no trabalho, carregando objetos (mais pesado que uma garrafa refri 2L)

			Nenhum
			Menos que 30 minutos por dia
			De 30 minutos a 1 hora por dia
			De 1 hora a 2 horas por dia
			De 2 hora a 3 horas por dia
			De 3 horas ou mais por dia

31 Em pé ou caminhando lentamente no trabalho não carregando nada.

			Nenhum
			Menos que 30 minutos por dia
			De 30 minutos a 1 hora por dia
			De 1 hora a 2 horas por dia
			De 2 hora a 3 horas por dia
			De 3 horas ou mais por dia

32 Caminhando rapidamente no trabalho, carregando objetos (mais pesado do que uma garrafa de refrig. 2L)

			Nenhum
			Menos que 30 minutos por dia
			De 30 minutos a 1 hora por dia
			De 1 hora a 2 horas por dia
			De 2 hora a 3 horas por dia
			De 3 horas ou mais por dia

33 Caminhando rapidamente no trabalho, não carregando nada.

			Nenhum
			Menos que 30 minutos por dia
			De 30 minutos a 1 hora por dia
			De 1 hora a 2 horas por dia
			De 2 hora a 3 horas por dia
			De 3 horas ou mais por dia
