# Supplementary material for: Do the body mass index and the diagnosis of gestational diabetes mellitus influence the level of physical activity during pregnancy and postpartum?
Source: PLoS One. 2019 Aug 9;14(8):e0220947. doi: 10.1371/journal.pone.0220947 (PMC6688803; doi:10.1371/journal.pone.0220947)
Supplement: S3 Appendix — Brazilian economic Classification Criteria from Associação Brasileira de Empresas e Pesquisas (Brazilian Association of Companies and Research). (PDF) [file pone.0220947.s003.pdf]

Data de Aplicação: ____/____/____

1. **Identificação**

Nome da Participante: ____

Número do prontuário: ____

Endereço: ____

Telefones de contato: ____________________ e _________________________

2. **Dados epidemiológicos**

Idade: anos

Etnia: (1) Branca (0) Não branca

Atividade Profissional: (1) do lar ; (2) trabalha fora; (3)estudante

Idade dos filhos (se tiver):

Estado Civil: casada (1) sim (0)não

Grau de escolaridade

(0) Analfabeta

(1) Ensino Fundamental Incompleto

(2) Ensino Fundamental Completo

(3) Ensino Médio Incompleto

(4) Ensino Médio Completo

(5) Superior Completo

Profissão: CBO:

Dados obstétricos

Idade Gestacional: ________ semanas ______ dias

Número de Gestações (incluindo a atual) : _____ Paridade: ______ Abortos: _____

Histórico Obstétrico Desfavorável? (1) sim (0) não

Gravidez Planejada? (1) sim (0) não

Possui antecedente psiquiátrico/ psicológico? (1) sim (0) não

Se sim, ainda faz acompanhamento? (1) sim (0) não

4. Critério de Classificação Econômica Brasil (CCEB)

Dados de classificação

**Itens de conforto**	**Não possui**	**1**	**2**	**3**	**4+**
Quantidade de automóveis de passeio exclusivamente para uso particular					
Quantidade de empregados mensalistas, considerando apenas os que trabalham pelo menos cinco dias por semana					
Quantidade de máquinas de lavar roupa, excluindo tanquinho					
Quantidade de banheiros					
DVD, incluindo qualquer dispositivo que leia DVD e desconsiderando DVD de automóvel					
Quantidade de geladeiras					
Quantidade de freezers independentes ou parte da geladeira duplex					
Quantidade de microcomputadores, considerando computadores de mesa, laptops, notebooks e netbooks e desconsiderando tablets, palms ou smartphones					
Quantidade de lavadora de louças					
Quantidade de fornos de micro-ondas					
Quantidade de motocicletas, desconsiderando as usadas exclusivamente para uso profissional					
Quantidade de máquinas secadoras de roupas, considerando lava e seca					

**A água proveniente deste domicilio é de:**	
1	Rede geral de distribuição
2	Poço ou nascente
3	Outro meio

**Considerando o trecho da rua de seu domicilio, você diria que a rua é:**	
1	Asfaltada/Pavimentada
2	Terra/Cascalho

Qual é o grau de instrução do chefe da família? Considere como chefe da família a pessoa que contribui com a maior parte da renda do domicílio

Nomenclatura atual	Nomenclatura anterior
Analfabeto/Fundamental I incompleto	Analfabeto/Primário incompleto
Fundamental I completo/ Fundamental II incompleto	Primário Completo/ Ginásio incompleto
Fundamental II completo/Médio incompleto	Ginásio Completo/Colegial incompleto
Médio completo/superior incompleto	Colegial completo/Superior incompleto
Superior completo	Superior completo

Classificação final: ________
